# P-2127. Early Post-Lung Transplant (LTx) Infections in the Antimicrobial Prophylaxis Era

**DOI:** 10.1093/ofid/ofaf695.2291

**Published:** 2026-01-11

**Authors:** Ashley N Estes, Adam M Ressler, Lizbeth Cahuayme-Zuniga, Marisa H Miceli

**Affiliations:** University of Michigan, Ann Arbor, MI; University of Michigan, Ann Arbor, MI; University of Michigan, Ann Arbor, MI; University of Michigan, Ann Arbor, MI

## Abstract

**Background:**

Despite widespread antimicrobial prophylaxis, infections remain a common complication after lung transplantation (LTx). This study aimed to identify the occurrence, risk factors, & outcomes of early infections post-LTx.

**Figure 1A:**
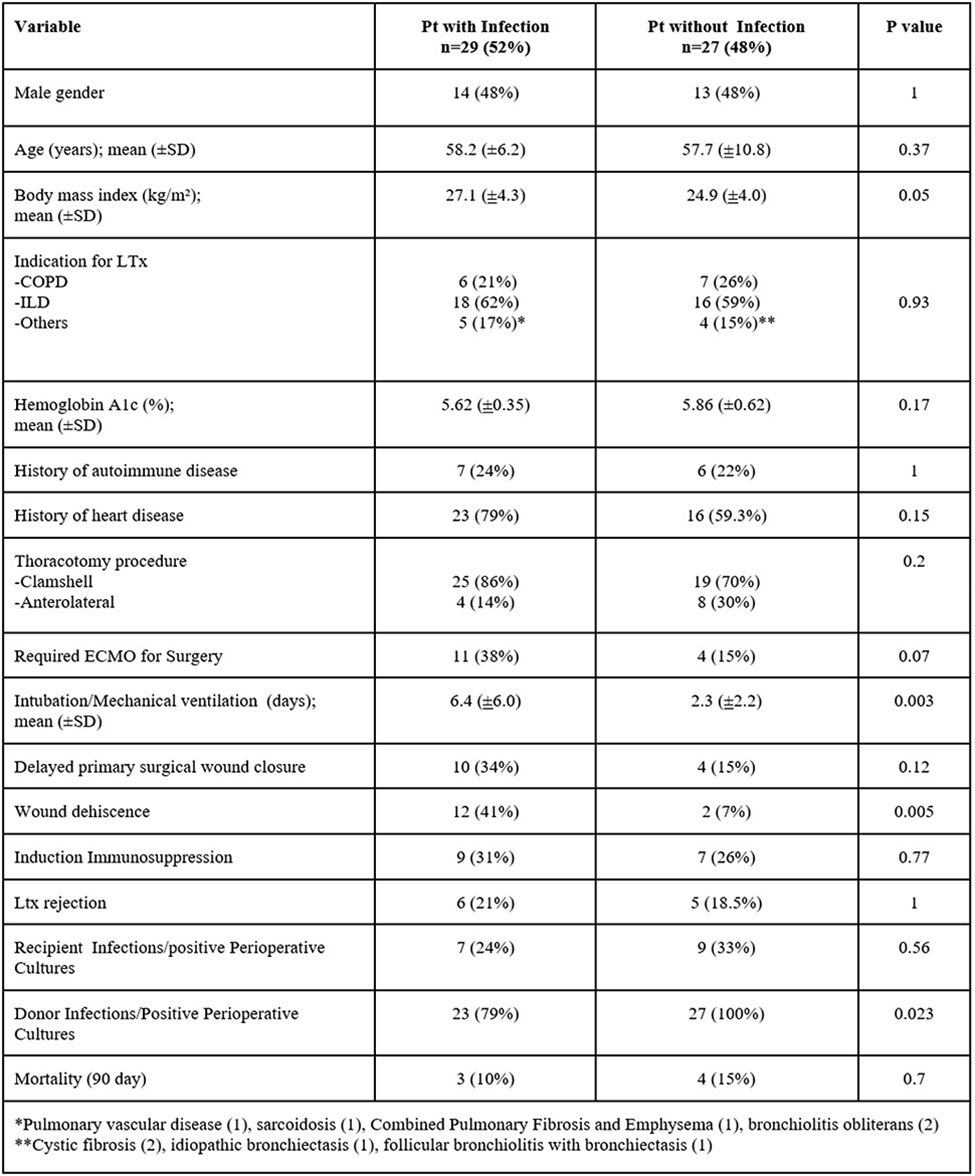
Infection Free Survival within 90 days after Lung transplantation

**Methods:**

We conducted a retrospective cohort study of adult LTx recipients at Michigan Medicine from 1/2021 to 4/2024. Demographics, surgical details, complications, immunosuppression, preoperative cultures, infections, & deaths within the first 90 days (d) after LTx were collected through chart review by a single reviewer & a second arbitrated unclear infection cases. Per institutional policy, all LTx pt received prophylaxis against surgical & opportunistic infections. A notification system for donor infections/colonization triggered targeted antimicrobials when needed. Fisher’s exact tests & Wilcoxon rank-sum tests analyzed categorical & continuous variables, respectively. Kaplan-Meier (K-M) curves assessed infection-free survival.

**Figure 1B:**
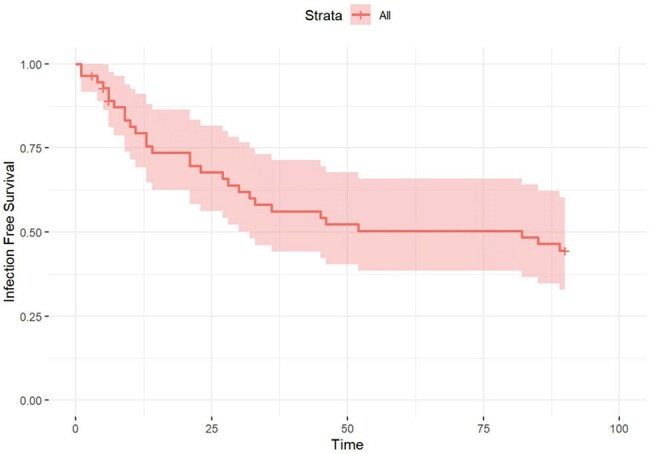
Infection Free Survival among lung transplant recipients who required ECMO and those without ECMO

**Figure:**
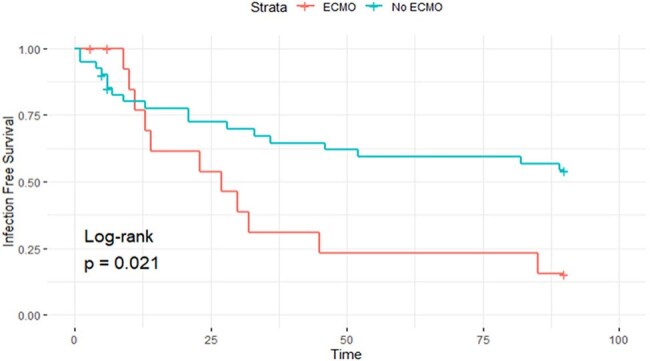

**Results:**

56 LTx recipients were included, 42 (75%) bilateral & 14 (25%) single LTx. Interstitial lung disease was the most common LTx indication (60.7%)(Table). Postoperative infections occurred in 29 pt (51.8%), 18 pt had 1 infection, 6 had 2, & 5 had≥3. Bacterial infections were most common (34; 66.7%), followed by viral (4; 7.8%) & fungal infections (3; 5.8%). Most common site of infection were the lungs [pneumonia (32), tracheobronchitis (6), empyema (4) & lung abscess (1)] & the surgical site (2). Increased infection risk was associated with longer intubation (6.4 vs. 2.3 d; p=0.003). Positive donor culture/infection was associated with a lower risk of infection given all pt without infection had positive donor cultures. Most infections occurred < 2 weeks after LTx, & more frequently among pt on ECMO (p=0.02) (Figure 1A&B). 7 pt died within 90 d after LTx, infection contributed to death in 2 pt.

**Conclusion:**

Bacterial infections, mainly pneumonia, were most common within 30 d after LTx, ECMO & longer intubation increased the risk of infection. These findings emphasize the need for ongoing evaluation & adjustment of infection prevention strategies, including efforts to reduce invasive procedures after LTx . Positive donor cultures did not increase the risk for infection, likely due to our ongoing notification system.

**Disclosures:**

Marisa H. Miceli, MD, F2G: PI in clinical trial|GSK: PI clinical trial|Pulmocide: PI in clinical trial|Scynexis: Advisor/Consultant

